# Cost-effectiveness of strategies to prevent road traffic injuries in eastern sub-Saharan Africa and Southeast Asia: new results from WHO-CHOICE

**DOI:** 10.1186/s12962-018-0161-4

**Published:** 2018-11-20

**Authors:** Ambinintsoa H. Ralaidovy, Abdulgafoor M. Bachani, Jeremy A. Lauer, Taavi Lai, Dan Chisholm

**Affiliations:** 1 Information, Evidence and Research, World Health Organization, Geneva, Switzerland; 2 Johns Hopkins International Injury Research Unit, Health Systems Program, Department of International Health, Johns Hopkins Bloomberg School of Public Health, Baltimore, MD USA; 3 Health Systems Governance and Financing, World Health Organization, Geneva, Switzerland; 4 Country Office, World Health Organization, Kyiv, Ukraine; 5 Mental Health and Mental Disorders, World Health Organization, Copenhagen, Denmark

**Keywords:** Cost-effectiveness analysis, Road traffic injury, Road safety, Priority setting, Resource allocation, Expansion path, WHO-CHOICE, Abdulgafoor m. bachani, Dan chisholm

## Abstract

**Background:**

Road safety has been receiving increased attention through the United Nations Decade of Action on Road Safety, and is also now specifically addressed in the sustainable development goals 3.6 and 11.2. In an effort to enhance the response to Road Traffic Injuries (RTIs), this paper aims to examine the cost effectiveness of proven preventive interventions and forms part of an update of the WHO-CHOICE programme.

**Methods:**

Generalized cost-effectiveness analysis (GCEA) approach was used for our analysis. GCEA applies a null reference case, in which the effects of currently implemented interventions are subtracted from current rates of burden, in order to identify the most efficient package of interventions. A population model was used to arrive at estimates of intervention effectiveness. All heath system costs required to deliver the intervention, regardless of payer, were included. Interventions are considered to be implemented for 100 years. The analysis was undertaken for eastern sub-Saharan Africa and Southeast Asia.

**Results:**

In Southeast Asia, among individual interventions, drink driving legislation and its enforcement via random breath testing of drivers at roadside checkpoints, at 80% coverage, was found to be the most cost-effective intervention. Moreover, the combination of “speed limits + random breath testing + motorcycle helmet use”, at 90% coverage, was found to be the most cost-effective package. In eastern sub-Saharan Africa, enforcement of speed limits via mobile/handheld cameras, at 80% coverage, was found to be the most cost-effective single intervention. The combination of “seatbelt use + motorcycle helmet use + speed limits + random breath testing” at 90% coverage was found to be the most cost-effective intervention package.

**Conclusion:**

This study presents updated estimates on cost-effectiveness of practical, evidence-based strategies that countries can use to address the burden of RTIs. The combination of individual interventions that enforces simultaneously multiple road safety measures are proving to be the most cost-effective scenarios. It is important to note, however, that, in addition to enacting and enforcing legislation on the risk factors highlighted as part of this paper, countries need to have a coordinated, multi-faceted strategy to improve road safety.

**Electronic supplementary material:**

The online version of this article (10.1186/s12962-018-0161-4) contains supplementary material, which is available to authorized users.

## Background

Annually, 1.25 million people die in road crashes worldwide [1]. Road traffic injuries (RTIs) represent the tenth leading cause of death among all age groups [2], and are predicted to be the seventh leading cause of death by 2030 [1]. RTIs are the leading cause of death among persons aged 15–29 years [1], and pedestrians, bicyclists, and motorcyclists represent 49% of all road traffic deaths [1]. The African region has the highest rates of road traffic deaths. RTIs are not only a public health problem, but also a development issue. As a result of RTIs, it has been estimated that low and middle-income countries (LMICs) lose approximately 3% of their gross domestic product (GDP) each year [1]. In recognition of the scale of the problem, road safety has been receiving increased attention through the United Nations Decade of Action on Road Safety, and it is also now specifically addressed in two of the sustainable development goals (SDGs). SDG target 3.6 calls for halving the number of global deaths and injuries from road traffic accidents by 2020 [3].

In an effort to enhance the response to RTIs, this paper aims to examine the cost effectiveness of proven interventions. This work forms part of an update of the WHO-CHOICE programme. Generalized cost-effectiveness analysis (GCEA) is used, which enables the efficiency of current interventions to be assessed alongside that of new interventions [4]. All currently recommended interventions are included in the analysis individually, and then as packages of care, based on combining the most cost-effective interventions.

For the purposes of consistency and comparability, this paper largely adopts the framework of an earlier WHO-CHOICE analysis [5, 6]. That analysis concluded that combined enforcement strategies represent the most efficient way to reduce the burden of RTIs, since combinations benefit from synergies on the cost side while producing greater overall health gain. This new analysis builds on that earlier work by using updated attributable fractions of RTIs associated with the different road users groups (pedestrians, bicyclists, car occupants, etc.) for our regions of interest, also by extending the time horizon of implementation from 10 years to 100 years. The following were also updated: the prevalence and distribution of RTIs (both fatal and non-fatal), the population sizes and mortality rates, the health-state valuations for long-term sequelae of RTIs, as well as the prices of the resources used in interventions.

## Methods

Detailed descriptions of the methods employed in WHO-CHOICE have been published previously [4, 7]. The goal of WHO-CHOICE is to compare both current and new interventions in terms of cost effectiveness. In this paper, we describe specific methods related to RTIs. The base year of 2010 was selected to be in line with the 2010 Global Burden of Disease study [8], whose data form the base of many of the disease models used in WHO-CHOICE. The analysis was undertaken for the eastern sub-Saharan Africa and Southeast Asia regions [9].

To allow for comparison of results in a sector-wide analysis, the WHO-CHOICE project evaluates interventions across a range of diseases and risk factors, using common methods. Health outcomes are measured as the gain in healthy life years (HLYs) due to an intervention. The use of HLYs allows for priority setting across the health sector since it facilitates comparison across different diseases. HLYs are reported both discounted at 3% per annum and undiscounted. WHO-CHOICE adopts the costing perspective of “the health system”, by which is meant the ensemble of actions and actors whose primary intent is to improve human health. The analysis, therefore, contains all direct, market-valued costs, whether public or private, that are required to deliver the intervention, regardless of payer. All costs are discounted at 3% per annum. Interventions are considered to be implemented for 100 years.

### Identification of risk factors and interventions for road traffic injuries

As for the previous WHO-CHOICE analysis, a dynamic system modelled with a Haddon matrix [10] was used as a reference framework for identifying factors that have an impact on RTI. Each cell of the matrix allows opportunities for an intervention to reduce road traffic injuries. Factors in italics are those included in the analysis (see Table 1).

**Table 1:** The Haddon matrix

Phase	Factors		
	Human	Vehicle	Environment
Pre-crash			
Crash prevention	Information Attitudes *Impairment* *Police enforcement*	Roadworthiness Lighting Braking Handling Speed management	Road design Road layout *Speed limits* Pedestrian facilities
Crash			
Injury prevention during the crash	*Use of restraints* *Impairment*	Occupant restraints Other safety devices Crash-protective design	Forgiving roadside
Post-crash			
Life sustaining	First-aid skill Access to hospital	Ease of access Fire risk	Rescue facilities Congestion

Source: World report on road traffic injury prevention, Fig. 1.3; factors in italics are those included in the analysis

This analysis evaluates 13 individual and combination interventions. They are drawn from recommendations in the the World report on road traffic injury prevention [10] and are mainly focused on pre-event road safety measures, targeting change in human behaviour, due to the availability of robust evidence on their effectiveness and feasibility (see Table 2).

**Table 2:** Interventions included in the analysis

#	Scenario name	Intervention	Description
1	RBT	Random breath testing	Drink driving legislations and its enforcement via random breath testing of drivers at roadside checkpoints
2	ESL	Enforcement of speed limits	Sustained effort by traffic enforcement teams to raise the perceived risk of drivers being caught via the use of mobile/hand held speed cameras at randomly chosen checkpoint sites
3	HUB	Bicycle helmet use	Legislation and enforcement of helmet use by bicyclists aged 15 years or less
4	HUM	Motorcycle helmet use	Legislation and enforcement of helmet use among riders of moped and motorcycles
5	SBU	Seatbelt use	Legislation and enforcement of seat belt use in cars (drivers and passengers)
6	SBU_HUM	Seatbelt use + motorcycle helmet use	
7	SBU_HUM_RBT	Seatbelt use + motorcycle helmet use + random breath testing	
8	SBU_HUM_ESL	Seatbelt use + motorcycle helmet use + enforcement of speed limits	
9	SBU_HUM_ESL_RBT	Seatbelt use + motorcycle helmet use + enforcement of speed limits + random breath testing	
10	SBU_HUM_ESL_RBT_HUB	Seatbelt use + motorcycle helmet use + enforcement of speed limits + random breath testing + bicycle helmet use	
11	ESL_RBT	Enforcement of speed limits + random breath testing	
12	ESL_RBT_HUM	Enforcement of speed limits + random breath testing + motorcycle helmet use	
13	ESL_RBT_SBU	Enforcement of speed limits + random breath testing + seatbelt use	

Key parameters in this analysis were the prevalence and distribution of RTIs, both fatal and non-fatal, the prevalence and distribution of risk factors for RTIs, the prevalence, distribution and effectiveness of interventions to reduce RTIs, the population size and mortality rates, and the health state valuations for the long-term sequelae of RTIs.

### Attribution of RTIs by road user group

**Fig. 1:**
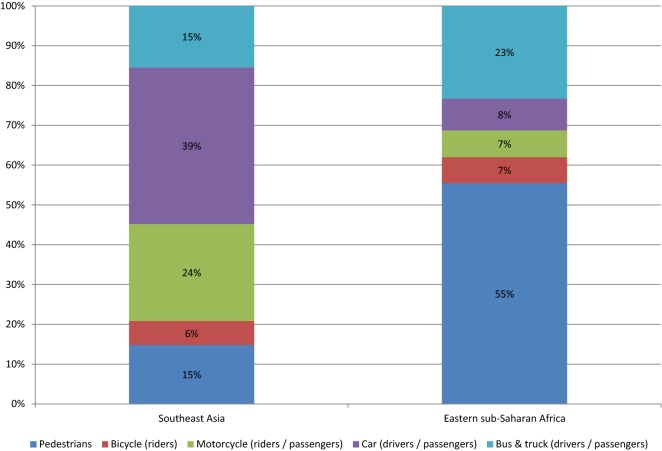
Distribution of road traffic fatalities by road user type calculated based on data provided by the International Injury Research Unit of Johns Hopkins Bloomberg School of Public Health

**Fig. 2:**
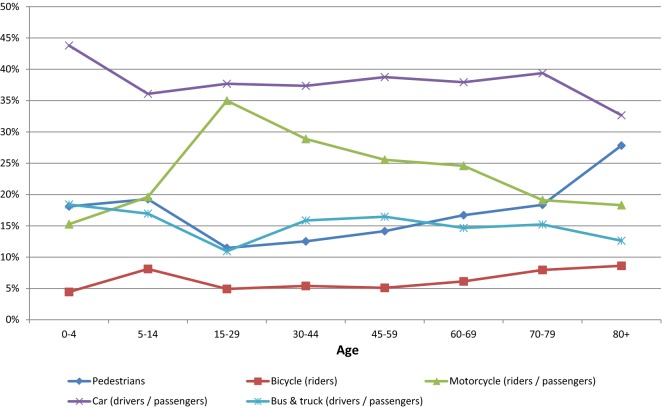
Age distribution of fatalities by road user type in Southeast Asia. Calculated based on data provided by the International Injury Research Unit of Johns Hopkins Bloomberg School of Public Health

**Fig. 3:**
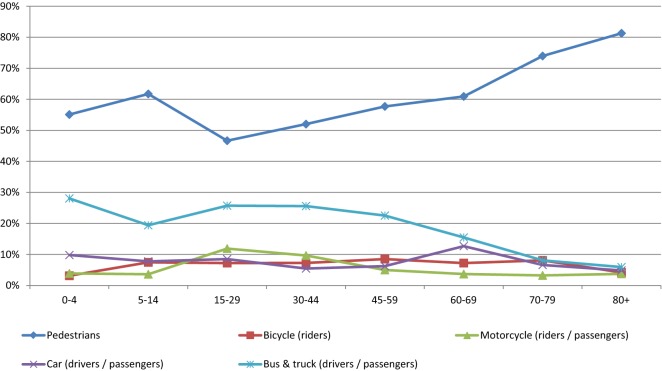
Age distribution of fatalities by road user type in Eastern sub-Saharan Africa. Calculated based on data provided by the International Injury Research Unit of Johns Hopkins Bloomberg School of Public Health

A literature review to give an overview of published data between 2006 and 2014 on fatal and non-fatal road traffic injuries, their risk factors and sequelae was conducted (see Additional file 1). The attributable fractions are calculated separately for all risk factors at the regional level based on the epidemiological evidence (e.g. exposure rates) from the countries in the region, weighted by population size. Key data on fatal and non-fatal injuries by road user type, sex and age group was provided by the International Injury Research Unit of the Johns Hopkins Bloomberg School of Public Health, which maintains and develops a global database of RTIs. Information collected with the literature review was used in triangulation of the attribution of the RTIs by road user group in combination of the data provided by the Johns Hopkins Bloomberg School of Public Health and the findings of the original literature review that informed the original model creation along with its attribution distribution (Figs. 1, 2, 3).

### Attribution of RTIs by risk factor

To measure the independent contribution of different risk factors to overall rates of RTIs in the population, we used the population attributable fraction (PAF), which can be defined as the fraction of incident cases attributable to the risk exposure:

$${\text{PAF}}\, = \,\frac{{\left( {{\text{Incidence}}\;{\text{of}}\;{\text{injury}}\;{\text{in}}\;{\text{all}}\;{\text{road}}\;{\text{users}}} \right) - \left( {{\text{Injury}}\;{\text{in}}\;{\text{road}}\;{\text{users}}\;{\text{without}}\;{\text{the}}\;{\text{exposure}}} \right)}}{{{\text{Incidence}}\;{\text{of}}\;{\text{injury}}\;{\text{in}}\;{\text{all}}\;{\text{road}}\;{\text{users}}}}.$$

### Estimation of intervention effectiveness

**Fig. 4:**
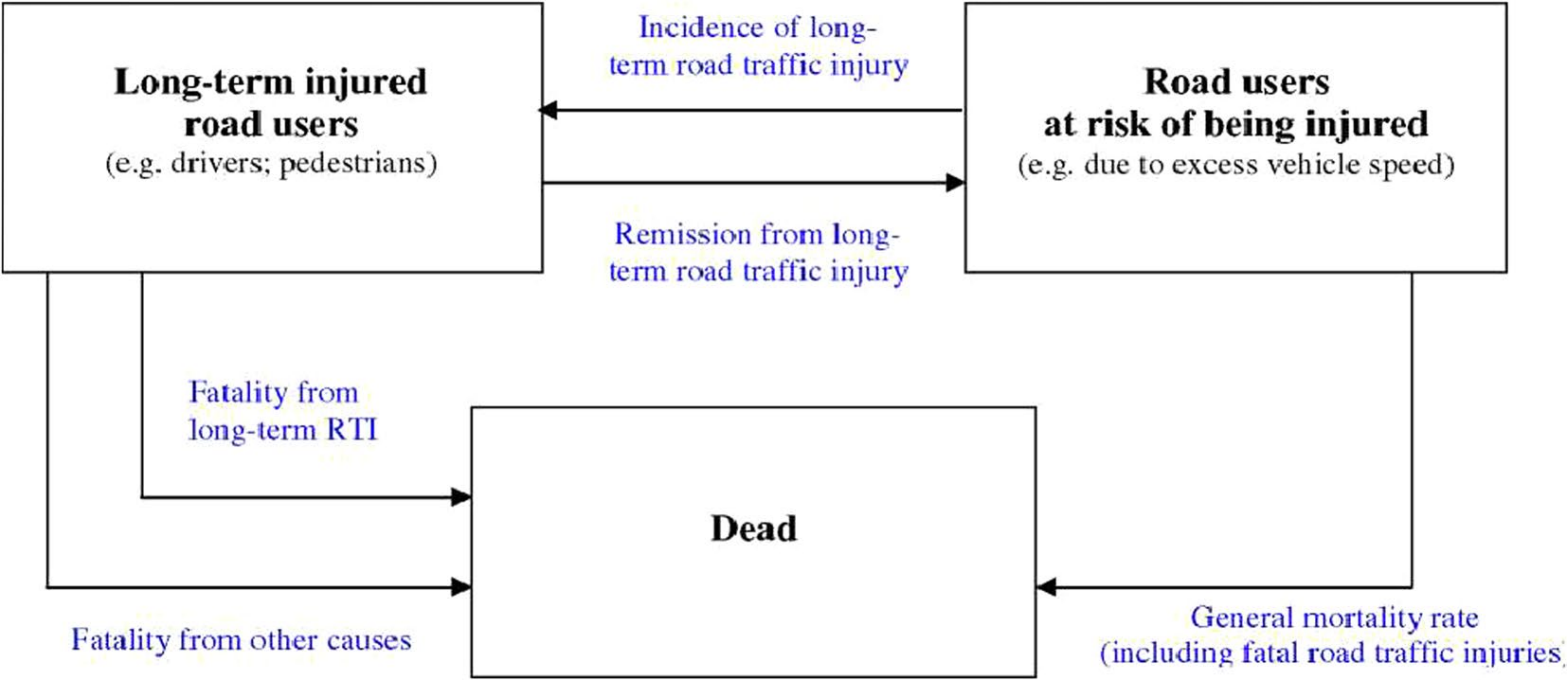
Population model for estimating health impact of road safety measures (Source: Road traffic injury prevention: an assessment of risk exposure and intervention cost effectiveness in different world region, 2008 [5], Fig. 8)

Interventions are at first compared to a hypothetical scenario where the known effects of implemented interventions are removed, referred to as the null scenario. Then the marginal impacts of interventions are evaluated with reference to the null scenario. A multi-state population model [11] was used to estimate scenarios (see Fig. 4). Further details on the methods can be found in [5]. Non-fatal acute injuries of short term duration (e.g. bruises, cuts) were not considered in the analysis.

The same estimates of the effects of interventions as in the previous WHO analysis [5] were used (see Additional file 2). This is due to the fact that during initial literature scoping on the intervention effects in the regions modelled, no papers of suitable focus and/or quality were found to enable updating of the sub-model of the intervention effect estimates in the targeted countries. The estimates used in this analysis of the incidence, prevalence and case fatality rates of RTIs, as well as their associated levels of disability are also shown in Additional file 2. The impact of the selected interventions on population health were evaluated individually, and then as a combination by multiplying the effects of each individual intervention.

### Intervention costing

Costs of interventions were estimated at the health system level, and include the costs of all market-valued inputs required to deliver the intervention. For example, costs include those of the passage of legislation, the enforcement of legislation and programme management [12]. For “bicycle helmet use” and “motorcycle helmet use” interventions, the costs of equipping bicyclists and motorcyclists with helmets were included, since these costs represent an integral component of those interventions. For the “seatbelt use” intervention, the costs of installing driver and passengers seatbelts in cars not already so equipped were included. Costs are discounted at 3% per annum, assuming a 100 year implementation period. Capital costs are annualized over the lifetime of the asset. All prices are in 2010 International Dollars. 2010 was chosen as the baseline year in line with the 2010 Global Burden of Disease epidemiological data which forms the base of many of the disease models used in WHO-CHOICE. The main costing assumptions are shown in Additional file 2.

## Results

The results for each intervention individually, and then as a package, are presented in Tables 3 and 4.

**Table 3:** Costs, effects and cost effectiveness of road safety measures in Southeast Asia over 100 years

Intervention (legislation and enforcement)	Pop° coverage (%)	Total costs per 10 million population (I$ 2010)	Healthy life years (HLY) gained per 10 million population	ACER (I$ per HLY)	ICER (I$ per HLY)
Random breath testing	80	117,632,481	52,288	2250	Dominated
Enforcement of Speed limits	80	120,598,909	44,216	2727	Dominated
Bicycle helmet use	80	111,809,164	1068	104,648	Dominated
Motorcycle helmet use	90	169,026,306	51,497	3282	Dominated
Seatbelt use	50	102,206,381	12,058	8476	Dominated
Seatbelt use + motorcycle helmet use	90	185,043,479	63,644	2907	Dominated
Seatbelt use + motorcycle helmet use + random breath testing	90	204,664,782	116,168	1762	Dominated
Seatbelt use + motorcycle helmet use + enforcement of speed limits	80	202,251,594	108,096	1871	Dominated
Seatbelt use + motorcycle helmet use + enforcement of speed limits + random breath testing	90	224,072,895	160,738	1394	1 552
Seatbelt use + motorcycle helmet use + enforcement of speed limits + random breath testing + bicycle helmet use	90	249,482,034	161,811	1542	23,692
Enforcement of speed limits + random breath testing	80	139,450,546	96,620	1443	Dominated
Enforcement of speed limits + random breath testing + motorcycle helmet use	90	205,065,577	148,493	1381	1381
Enforcement of speed limits + random breath testing + seatbelt use	80	158,109,184	108,774	1454	Dominated

**Table 4:** Costs, effects and cost effectiveness of road safety measures in Eastern sub-Saharan Africa over 100 years

Intervention (legislation and enforcement)	Pop° coverage (%)	Total costs per 10 million population (I$ 2010)	Healthy life years (HLY) gained per 10 million population	ACER (I$ per HLY)	ICER (I$ per HLY)
Random breath testing	80	371,264,947	8242	45,048	Dominated
Enforcement of speed limits	80	372,557,382	14,576	25,559	Dominated
Bicycle helmet use	80	367,527,956	243	1,514,136	Dominated
Motorcycle helmet use	90	385,934,475	6191	62,343	Dominated
Seatbelt use	50	336,588,617	3480	96,715	Dominated
Seatbelt use + motorcycle helmet use	90	439,366,375	9688	45,353	Dominated
Seatbelt use + motorcycle helmet use + random breath testing	90	495,706,294	17,972	27,583	Dominated
Seatbelt use + motorcycle helmet use +enforcement of speed limits	80	485,490,048	24,335	19,950	Dominated
Seatbelt use + motorcycle helmet use + enforcement of speed limits + random breath testing	90	551,981,331	32,649	16,907	16,907
Seatbelt use + motorcycle helmet use + enforcement of speed limits + random breath testing + bicycle helmet use	90	612,222,569	32,892	18,613	247,240
Enforcement of speed limits + random breath testing	80	427,607,093	22,846	18,717	Dominated
Enforcement of speed limits + random breath testing + motorcycle helmet use	90	496,182,560	29,060	17,074	Dominated
Enforcement of speed limits + random breath testing + seatbelt use	80	482,432,030	26,417	18,262	Dominated

### Population-level effects of interventions

The effectiveness of interventions are reported in healthy life years (HLYs) gained due to the specific intervention (Tables 3 and 4).

Because the highest road fatalities are among car drivers and passengers in Southeast Asia (39% of all fatalities, Fig. 1), drink driving legislation and its enforcement via “random breath testing” at roadside checkpoints was found to be the most effective single intervention in this region. The legislation “motorcycle helmet use”, and its enforcement, was found to be the second most effective single intervention; this is consistent with the high proportion of motorcycles in this region and the percentage of road fatalities among this road user group (24%, Fig. 1).

In eastern sub-Saharan Africa, the enforcement of “speed limits” via mobile/handheld cameras at 80% coverage was found to be the most effective single intervention, probably reflecting the fact that pedestrians account for more than 50% of road fatalities among all road user groups in this region (see Fig. 1).

The legislation and enforcement of “bicycle helmet use”, at 80% coverage, was found to be the least effective single intervention in both regions.

Among the combination of interventions, a scenario that combined all five individual interventions was found to be the most effective in both regions.

### Population level costs of interventions

The total costs estimated for motorcycle helmet use include not only the costs of the passage of legislation and its enforcement but also the costs to the household of purchasing safety equipment, which may explain why this intervention represents the most costly single intervention in both sub-regions. The household cost component is also added to the costs of “seatbelt use” and “bicycle helmet use”; the costs of “seatbelt use” is applied to cars that are not already equipped and “bicycle helmet use” targets only children aged 15 years or less (Tables 3 and 4).

Economies of scope are realised by combining individual interventions due to the synergies that exists between different enforcement strategies.

### Cost effectiveness of interventions

The cost effectiveness of individual interventions and their combinations are presented in Tables 3 and 4. Cost-effectiveness ratios are reported as costs (in international dollars) per HLY gained.

Among single interventions, “random breath testing”, at 80% coverage, was found to be the most cost-effective intervention in Southeast Asia, whereas in eastern sub-Saharan Africa, it was “speed limits”, at 80% coverage.

Combinations of individual interventions were found to be the most cost-effective: “speed limits + random breath testing + motorcycle helmet use”, at 90% coverage, in Southeast Asia and “seatbelt use + motorcycle helmet use + speed limits + random breath testing”, at 90% coverage, in eastern sub-Saharan Africa.

**Fig. 5:**
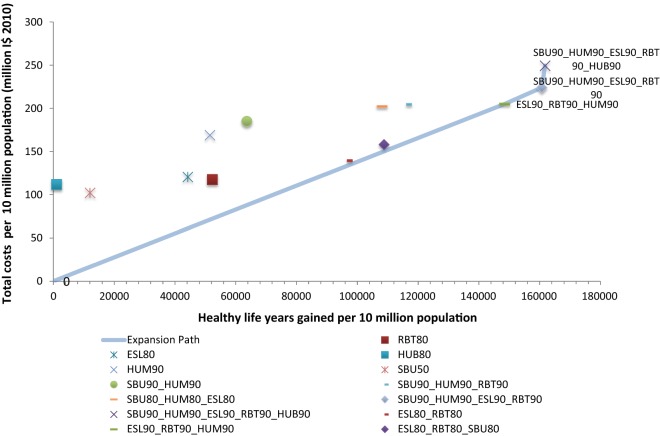
Cost-effectiveness expansion path for Southeast Asia. Refer to Table 2 for interventions’ labels

**Fig. 6:**
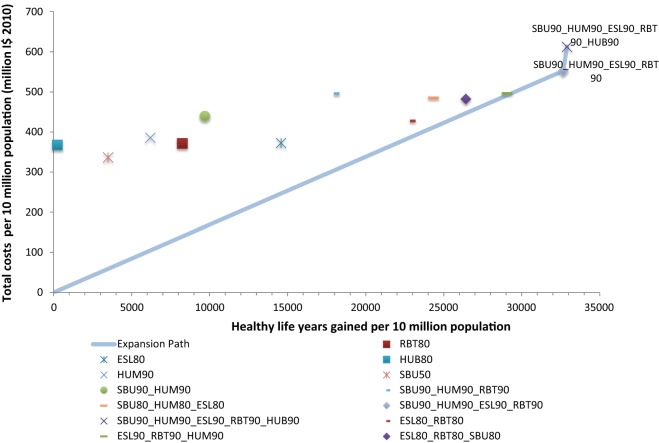
Cost-effectiveness expansion path for Eastern sub-Saharan Africa. Refer to Table 2 for interventions’ labels

Figures 5 and 6 show the expansion path a decision maker could follow to achieve the maximum health gain for a given level of expenditure. The expansion path shows the order in which each intervention would be adopted based on its incremental cost-effectiveness ratio, compared to the previously adopted intervention, until no more health gain is possible [4].

Following the expansion path in Fig. 5, in Southeast Asia policymakers would first implement “speed limits + random breath testing + motorcycle helmet use”, at 90% coverage, and when additional resources become available, add “seatbelt use”, at 90% coverage, followed by “bicycle helmet use”, also at 90% coverage.

In eastern sub-Saharan Africa, after “seatbelt use + motorcycle helmet use + speed limits + random breath testing”, at 90% coverage, a policymaker could add “bicycle helmet use”, also at 90% coverage, to maximize health gain (see Fig. 6).

## Discussion

This paper adopts the framework of the 2012 study and is showing that the most cost effective interventions are essentially unchanged. However, the ranking of interventions is slightly different. Bicycle helmet use, while being on the expansion path (as a single intervention) in the previous analysis for countries in sub-Saharan Africa, is now shown to be less cost effective in this update unless combined with other interventions. The combination of speed limits, random breath testing and motorcycle helmet use at 90% coverage also appears on the expansion path in this update, and is the most cost effective combination of interventions in Southeast Asia, while it was dominated in the previous analysis. Nevertheless, these findings corroborate the conclusion of the previous analysis stating that combined enforcement strategies represent the most efficient way to reduce the burden of RTIs.

The analysis presented in this paper underscores the cost-effective nature of interventions to prevent road traffic injuries in low-income and lower middle-income countries. As previous studies have demonstrated, compared to other public health measures, strategies to improve road safety are cost-effective interventions [6, 13–15]. Our analysis shows that interventions aimed at enforcing legislation for road safety are especially effective, as they improve cost efficiencies while also enhancing gains in effectiveness.

The interventions included in our analysis are in line with the recently proposed Save-LIVES technical package published by WHO [16]. This package was developed to provide a comprehensive, evidence-based set of tools to address the growing burden of RTIs globally. Based on the recommendations included in this package, legislation and its enforcement are the cornerstones of an effective road safety programme. Our findings, which show significant potential gains as a result of enacting and enforcing legislation targeting the leading risk factors for road traffic injuries, support this recommendation.

As the United Nations Decade of Action for Road Safety reaches its final years, and with the goal of halving the world’s road traffic deaths by the year 2020 (SDG 3.6) upon us, there is an increased sense of urgency to address the burden of RTIs globally [3, 17]. Action needs to be taken at national levels, and countries should identify and implement strategies to improve road safety within their borders. In recognition of the fact that policy-makers work under resource-constrained conditions, and have to make decisions about competing programs, our analysis presents a practical approach that identifies the most cost-effective individual interventions that countries could implement first, followed by an expansion strategy that can be employed as more resources become available. Such a phased approach is more likely to be more feasible than an all-or-nothing option.

A limitation of our analysis is that we take a regional perspective, rather than a country specific one, and that we present analysis for only two regions in the world. These are, however, regions that have high burdens of RTIs and related fatalities. It is also expected that the findings would hold true at country level.

## Conclusion

This study presents updated estimates on cost-effectiveness of practical, evidence-based strategies that countries can use to address the burden of RTIs. It is important to note, however, that, in addition to enacting and enforcing legislation on the risk factors highlighted as part of this paper, countries need to have a coordinated, multi-faceted strategy to improve road safety that includes leadership and coordination of activities around road safety; efficient and reliable mechanisms to gather data that would aid in understanding the burden as well as evaluating the effectiveness and efficiency of programs; infrastructural improvements; a focus on vehicle safety standards; and a coordinated post-crash care system that is aimed at minimizing the impact of a road accident on the individual.

## Additional files

**Additional file 1.** Detailed results of the literature review (2006–2014).

**Additional file 2.** Effect sizes and costing assumptions.

## Abbreviations

ACER: average cost-effectiveness ratio
CHOICE: CHOosing Interventions that are Cost-Effective
GCEA: generalized cost-effectiveness analysis
GDP: gross domestic product
HLY: healthy life years
ICER: incremental cost-effectiveness ratio
PAF: population attributable fraction
RTI: road traffic injuries
SDGs: sustainable development goals
WHO: World Health Organization
