# Supplementary material for: Cost-effectiveness of strategies to prevent road traffic injuries in eastern sub-Saharan Africa and Southeast Asia: new results from WHO-CHOICE
Source: Cost Eff Resour Alloc. 2018 Nov 20;16:59. doi: 10.1186/s12962-018-0161-4 (PMC6245850; doi:10.1186/s12962-018-0161-4)
Supplement: Supplementary file 1 — Additional file 1. Detailed results of the literature review (2006–2014). [file 12962_2018_161_MOESM1_ESM.docx]

# Cost-effectiveness of strategies to prevent road traffic injuries in eastern sub-Saharan Africa and Southeast Asia: new results from WHO-CHOICE

# Additional file 1: Detailed results of the literature review (2006-2014)

## Introduction

The aim of this literature review is to give an overview of recently published data on fatal and non-fatal road traffic injuries, their risk factors and sequelae in Sub-Saharan Africa and South-East Asia for renewing the model of cost-effectiveness of road traffic safety interventions.

Published studies and unpublished reports on country-specific road traffic injuries, their sequelae and road user distribution from 2006 and later (that were not used in the previous report) were sought by:

- An online keyword search using search engines such as EBSCO (incl. Medline), Google Scholar, Google, PubMed
- Relevant references cited in articles identified by the electronic search and relevant articles referring to identified articles were selected

The number of peer-reviewed articles reporting population-based distributions of road traffic injuries by road user category was limited; the majority of the data sources were mostly urban, hospital-based studies. More than one data source was identified for a number of countries.

Some articles are cited under more than one topic, if they include data on both.

## 1. Age- and sex-specific road traffic fatality rates

For attributing total injury estimates across different age groups, we tried to find a sub-set of countries that provided this detailed level of information (data were found for South Africa, Tanzania, India, Thailand). Last (2008) report by Chisholm & Naci found that the overall age distribution for fatalities and non-fatal injuries by road user type does not differ greatly among countries, although South Africa is at variance with the other countries due to a much lower life expectancy in age groups over 60.

Data on fatal road traffic injuries were more widespread than for non-fatal injuries. Very few studies provided a detailed distribution of road traffic fatalities or injuries by sex and age group, and more detailed age groups were available in very few studies.

### *1.1 Age- and sex specific RTI fatality rates in Sub-Saharan Africa*

For Sub-Saharan Africa region an age distribution of RTI fatalities was detected for 4 countries, a total of 10 articles. These studies are outlined in Table 1. Of these, data for Republic of South Africa, Tanzania, and Kenya provided a detailed distribution of RTI by age that was close to the distribution used in the previous study.

Most comprehensive statistics on RTI fatalities are available for South Africa where a mortality surveillance system is in place. For Kenya one hospital-based study was found, as well as one report based on data from the police, and one survey study. For Tanzania and Ethiopia survey data and a few hospital-based studies were found. In most of these studies road traffic fatality data were reported by age groups and by sex separately, so that a detailed age/sex distribution has to be imputed.

**Table 1.** Data sources for age- and sex-specific road traffic fatality rates in Sub-Saharan Africa

| **Reference** | **Country** | **Region** | **Type of data** | **Data period** | **n** | **Age groups reported** | **Findings** |
| --- | --- | --- | --- | --- | --- | --- | --- |
| Norman, R., Matzopoulos, R., Groenewald, P., & Bradshawa, D. (2007). The high burden of injuries in South Africa. *Bulletin of the World Health Organization 2007*(85), 695–702. | South Africa | - | surveillance | 2000 | 59 935 injury deaths | 0-4, 5-14, 15-29, 30-44, >60 | Rate per 100 000 by sex & age group |
| *A profile of fatal injuries in South Africa* - 7th Annual Report of the NATIONAL INJURY MORTALITY SURVEILLANCE SYSTEM 2005 | South Africa | - | surveillance | 2005 | 29 596 injury deaths, 5675 traffic deaths | <1, 1-4, 5-9, 10-14, 15-19, 20-24, 25-29, 30-34, 35-39, 40-44, 45-49, 50-54, 55-59, 60-64, 65+ | Absolute numbers by age group |
| Statistics South Africa (2009). *Road Traffic Accident Deaths in South Africa, 2001–2006: Evidence from death notification.* Report No. 03-09-07 (2001–2006). | South Africa | - | death notifications | 2001-2006 | 28 890 road traffic deaths | 0-14, 15-24, 25-34, 35-49, 50-64, 65+ | Deaths per 100 000 by sex & year, deaths per 100 000 by age & year, deaths per 100 000 by sex and age |
| Bachani, A. M., Koradia, P., Herbert, H. K., Mogere, S., Akungah, D., Nyamari, J., Osoro, E., Maina, W., & Stevens, K. A. (2012) Road Traffic Injuries in Kenya: The Health Burden and Risk Factors in Two Districts, *Traffic Injury Prevention, 13*(sup1), 24-30. | Kenya | Thika, Naivasha, urban + rural | traffic police, vital registration, observations | 2004-2009 | n/a | <1, 1-4, 5-14, 15-24, 25-34, 35-44, 45-54, 55-74, >74 | % of RTI fatalities by sex & age |
| Gichuhi , K. (2007). Injury Pattern Among Non-fatal Road Traffic Crash Victims. *East African Orthopaedic Journal 1. 23-25.* | Kenya | Nairobi, urban? | hospital-based study | 2004 | 1424 RTI victims treated in hospital | 0-4, 5-9, 10-14, 15-19, 20-24, 25-29, 30-34, 35-39, 40-44, 45-49, 50-54, 55-59, 60-64, 65-69, 70+ | Number of RTIs by age group |
| Macharia , W. M., Njeru, E. K., Muli-Musiime, F., & Nantulya, V. (2009). Severe road traffic injuries in Kenya, quality of care and access. *African Health Sciences 9*(2), 118-124. | Kenya | n/a | survey | 1998-1999 | 310 RTI casualties | <15, 15-24, 25-49, >49 | Number of RTIs by age group, number of RTIs by sex |
| Komba, D. D. (2006). *Risk Factors and Road Traffic Accidents in Tanzania: A Case Study of Kibaha District*. Master Thesis in Development Studies, Norwegian University of Science and Technology (NTNU) | Tan­zania | Kibaha | hospital data | 2001-2004 | 246 fatal, 591 non-fatal | 0-17, 18-24, 25-34, 35-44, 45+ | Number of fatal & non-fatal RTIs by sex & age group |
| Masaoe, E. N. (2007). Study on Road Accidents in Mainland Tanzania. Final Report submitted to Surface and Marine Transport Regulatory Authority (SUMATRA). http://www.sumatra.or.tz/index.php/component/docman/doc_view/49-study-on-road-accidents-in-mainland-tanzania?Itemid=317 (Accessed on 24.07.2014) | Tan­zania | Dar es Salaam, Coast, Arusha, Kilimanjaro | post-accident survey | 1994-2007 | 102 accident victims | <7, 8-12, 13-19, 20-24, 25-29, 30-34, 35-39, 40-44, 45-49, 50-54, >55 | Distribution of RTIs by age group & sex (approximate), fatal & nonfatal injuries by age group (total + percentage) |
| Zimmerman, K., Mzige, A. A., Kibatala, P. L., Museru, L. M., Guerrero, A. (2012). Road traffic injury incidence and crash characteristics in Dar es Salaam: A population based study. *Accident Analysis and Prevention 45*, 204– 210. | Tan­zania | Dar es Salaam | household survey | ? | 196 non-fatal RTI victims | 0–4, 5–14, 15–44, >45 | Number of RTIs by age group |
| Woldemichael, K., & Berhanu, N. (2011). Magnitude and pattern of injury in Jimma University specialized hospital, South-West Ethiopia. *Ethiopian Journal of Health Sciences 21*(3). 155-165. | Ethiopia | Jimma, South-West Ethiopia | hospital-based study | 2010-2011 | 334 RTA victims | 0-4, 5-14, 15-49, 50-64, >64 | Number of RTIs by age group, number of RTIs by sex |

### 1.2 Age- and sex specific RTI fatality rates in South-East Asia

For South-East Asia data for 4 countries were detected, a total of 12 articles with available data for India, Thailand, Vietnam and Nepal (see Table 2). Of these, some data for India and Thailand provided a detailed distribution of RTI by age, and couple of studies (e.g. Hsiao et al. 2013; Ditsuwan et al. 2011) attempted to correct for underreporting of RTIs and to fill in the data gaps with data from various sources.

For India, mostly hospital-based and autopsy studies were available, but one large mortality survey/verbal autopsy study (Hsiao et al. 2013) is probably the most comprehensive data source for RTI-related mortality in India, and provides good estimates.

For Thailand a comprehensive Burden of Disease study was found (Ditsuwan et al. 2011) and for Vietnam some quite comprehensive preliminary surveillance data (Ngo et al. 2012) representing 3% of the population was found. For Nepal only one hospital-based study was available, and for other countries in the region no recent data were found.

**Table 2.** Data sources for age- and sex-specific road traffic fatality rates in South-East Asia

| **Reference** | **Country** | **Region** | **Type of data** | **Data period** | **n** | **Age groups reported** | **Findings** |
| --- | --- | --- | --- | --- | --- | --- | --- |
| Dandona, R., Kumar, A., Ameer, A., Ahmed, M., & Dandona, L. (2008). Incidence and Burden of Road Traffic Injuries in Urban India. *Injury Prevention 14*(6), 354–359. | India | Hyderabad city | survey data | 2005-2009 | 536 non-fatal RTIs by 520 participants | 5-9, 10-14, 15-19, 20-29, 30-39, 40-49 | Estimated annual non-fatal RTI incidence rate per 100 persons in population aged 5-49 years |
| Honnungar, R. S., Aramani, S. C., Vijay Kumar, A. G., Ajay Kumar, T. S, Jirli, P. S. (2011). An Epidemiological Survey of Fatal Road Traffic Accidents and their Relationship with Head Injuries. *Journal of Indian Academic Forensic Medicine 33*(2), 135-137. | India | Karnataka | autopsy study | 2004-2009 | 506 vehicle accident fatalities | <10, 11-20, 21-30, 31-40, 41-50, 51-60, >60 | Fatal RTIs by age group & sex |
| Hsiao, M., Malhotra, A., Thakur, J.S., et al. (2013). Road traffic injury mortality and its mechanisms in India: nationally representative mortality survey of 1.1 million homes. *BMJ Open 2013(3*):e002621. | India |  | large mortality survey, verbal autopsy | 2001-2005 | 11543 injury deaths | 0-4, 5-14, 15-29, 30-44, 45-59, 60-69, >70 | Number of RTIs by sex and age group, estimated totals for 2005 |
| Kakeri, S. R., Bagali, M.A., Goudar, E.S., & Qadri, S. Y. (2014). Pattern of injuries and death sustained by the occupants of the two-wheeler during road traffic accidents. *Al Ameen Journal of Medical Science 7*(2), 118-124. | India | Bijapur | hospital-based study | 2005-2007 | 150 RTA victims | <10, 10-20, 20-30, 30-40, 40-50, 50-60, 60-70, >70 | Fatal RTIs by age group |
| Khajuria, B., Sharma, R., & Verma, A. (2008). A profile of the autopsies of road traffic accident victims in Jammu. *Journal of Clinical and Diagnostic Research 2*, 639-642 | India | Jammu | autopsy study | 2000-2005 | 249 RTA victims | <20, 20-40, 41-60, >60 | Fatal RTIs by age group, fatal RTIs by sex |
| Mohan Kumar, T.S., Tanuj Kanchan, Yoganarasimha, K., Pradeep Kumar, G. (2006). Profile of unnatural deaths in Manipal, Southern India 1994–2004. *Journal of Clinical Forensic Medicine 13*(3), 117-120. | India | Manipal, Southern India | autopsy study | 1994-2004 | 653 RTA victims | 0–9, 10–19, 20–29, 30–39, 40–49, 50–59, >60 | Fatal RTIs by age group & sex |
| Sharma, B.R., Sharma, A.K., Sharma, S. & Singh, H. (2007). Fatal Road Traffic Injuries in Northern India: Can They Be Prevented? *Trends in Medical Research 2*(3), 142-148. | India | Northern India | autopsy study | 1996-2005 | 1109 RTA victims | 0-10, 11-15, 16-20, 21-25, 26-30, 31-40, 41-50, 51-60, >61 | Fatal RTIs by age group & sex |
| Manish, K, Jyothi, N. S, Pawar, G. S., Jatti, V. B. (2012). Fatal Head Injuries in Road Traffic Accidents in and around Davangere: A Prospective Study. Indian Journal of Forensic Medicine and Pathology 5(2). | India | Davangere | hospital-based study | 2005-2007 | 408 RTI deaths | 0-10, 11-20, 21-30, 31-40, 41-50, 51-60, 61-70, 71-80 | Fatal RTIs by age group |
| Ditsuwan, V., Veerman, L. J., Barendregt, J. J., Bertram, M., & Vos, T. (2011). The national burden of road traffic injuries in Thailand. *Population Health Metrics 9*(2) | Thailand |  | estimate of fatal RTIs from SPICE cause of death study, hospital data, injury surveillance data, emergency department data | 2004 | 567000 RTI victims | 0-4, 5-14, 15-29, 30-44, 45-59, 60-69, 70-79, 80+ | Fatal & nonfatal RTIs: deaths, admissions and RTI victims at emergency departments by age group & sex |
| Nakahara, S., Chadbunchachai, W., Ichikawa, M., *Tipsuntornsak* N., Wakai, S. (2005). Temporal distribution of motorcyclist injuries and risk of fatalities in relation to age, helmet use, and riding while intoxicated in Khon Kaen, Thailand. *Accident Analysis and Prevention 37*, 833–842. | Thailand | Khon Khaen | hospital-based study | 1998-2002 | 9948 injured motorcyclists | 10-19, 20-29, 30-39, 40+ | Motorcycle RTIs by age group, motorcycle RTIs by sex |
| Ngo, A.D., Rao, C., Phuong Hoa, N., Hoy, D. G., Quynh Trang, K. T., & Hill, P. S. (2012). Road traffic related mortality in Vietnam: Evidence for policy from a national sample mortality surveillance system. *BMC Public Health 12*, 561. | Vietnam | Sample of 192 communes in 16 provinces, representing six socioeconomic regions in Vietnam (3% of pop) | surveillance data, verbal autopsy | 2008-2009 | 1061 RTA victims | <15, 15-19, 20-29, 30-39, 40-49, 50-59, 60+ | Number of RTI deaths by age group, number of RTI victims by sex |
| Mishra, B., Sinha, N. D., Suhkla, S. K., & Sinha, A. K. (2010). Epidemiological Study of Road Traffic Accident Cases from Western Nepal. *Indian Journal of Community Medicine 35*(1), 115–121. | Nepal | Western Nepal | hospital-based study |  | 360 RTA victims | 0-15, 16-30, 31-45, 46-60, >60 | RTA victims by age, RTA victims by sex |

## 2. Road users: age distribution, risk factors & injuries by road users

A standardized online keyword search was carried out to obtain country specific risk factor information, using online search engines such as Google, Google Scholar, and EBSCO. Keywords used were country name+road traffic injuries+road user, country name+ road traffic injuries+pedestrians, country name+road traffic injuries+motorcycle riders, country name+road traffic injuries+bicyclists, country name+road traffic injuries+car occupants, vehicle occupants.

Very few studies provided information on the distribution of non-fatal injuries by road user category. Additionally, very few studies provided age breakdowns or compared deaths in different road user groups by sex. Classification of casualties by category of road-users was not uniform and in many instances such aggregated groupings did not allow for accurate identification of road-user categories.

### 2.1 Age distribution of road users

As to age distribution of road users, the only available estimations were for South Africa. They originated from 2004, and provided an expected distribution of road users, based on calculations and data from 1997-1998 (Table 3).

**Table 3.** Data sources for age distribution of road users

| **Reference** | **Country** | **Region** | **Type of data** | **Data period** | **n** | **Age groups reported** | **Findings** |
| --- | --- | --- | --- | --- | --- | --- | --- |
| De Beer, E.J.H., & van Niekerk, E.C. (2004). *The estimation of unit costs of road traffic accidents in South Africa*. National Department of Transport Contract Report CR-2004/6 | South Africa |  | calculations | 1997-1998 | n/a | 0-1, 1-4, 5-9, 10-14, 15-19, 20-24, 25-29, 30-34, 35-39, 40-44, 45-49, 50-54, 55-59, 60-64, 65-69, 70-74, 75-59, 80+ | expected distribution of road users, based on calculations |

### 2.2 Distribution of risk factors by road users

Search keywords used were country name+seat belt, country name+helmet, country name+speeding, country name+drink-driving, driving under influence, alcohol-impaired driving.

The aim was to locate country-specific risk-factor information, not only relating to direct assessment of the contribution of specific risk factors to overall road traffic injury rates, but also relating to levels of risk factor exposure (e.g. not wearing seatbelts). Concerning direct measures, only a limited number of country specific references were found, mostly from journal articles based on police or hospital data.

Distributing road users into five distinct categories was problematic, because local classification systems included local means of transportation which have an arbitrary number of wheels and could be motorized or not, or powered by draft animals.

In some cases no distinction was made between motorized and non-motorized two-wheelers, in some cases bus & truck occupants were grouped together as motorized four-wheeler occupants. In some cases data for drivers and pillions were reported separately.

#### 2.2.1 Distribution of risk factors by road users in Sub-Saharan Africa

Data on risk factor distribution by road users were available for 6 countries from Sub-Saharan Africa, a total of 7 articles (Table 4). Risk factor data are notably fragmented, and distribution of risk factors by age groups was not reported in any study. Blood alcohol concentration is not routinely measured, and not using safety equipment is often not recorded in hospital data.

**Table 4.** Data sources on distribution of risk factors by road users in Sub-Saharan Africa

| **Reference** | **Country** | **Region** | **Type of data** | **Data period** | **n** | **Road user groups reported** | **Findings** |
| --- | --- | --- | --- | --- | --- | --- | --- |
| Bachani, A. M., Koradia, P., Herbert, H. K., Mogere, S., Akungah, D., Nyamari, J., Osoro, E., Maina, W., & Stevens, K. A. (2012) Road Traffic Injuries in Kenya: The Health Burden and Risk Factors in Two Districts, *Traffic Injury Prevention, 13*(sup1), 24-30. | Kenya | Thika, Naivasha, urban + rural | traffic police, vital registration, observations | 2010 | 6218 | all road users | Motorcycle drivers wearing a helmet - 30,37%, passengers 4,06 |
| Masaoe, E. N. (2007). Study on Road Accidents in Mainland Tanzania. Final Report submitted to Surface and Marine Transport Regulatory Authority (SUMATRA). http://www.sumatra.or.tz/index.php/component/docman/doc_view/49-study-on-road-accidents-in-mainland-tanzania?Itemid=317 (Accessed on 24.07.2014) | Tan­zania | Dar es Salaam, Coast, Arusha, Kilimanjaro | traffic police statistics | 2000-2005 | 85434 | all road users | Excessive speed 3,7%, reckless driving 54,5%, intoxication 0,8% of RTIs |
| Chalya, P. L., Mabula, J. B., Dass, R. M., Mbelenge, N., Ngayomela, I. H., Chandika, A. B., & Gilyoma, J. M. (2012). Injury characteristics and outcome of road traffic crash victims at Bugando Medical Centre in Northwestern Tanzania. *Journal of Trauma Management & Outcomes 6*(1). | Tan­zania | Northwestern Tanzania | hospital-based study | 2010-2011 | 1678 road traffic crash victims | all road users | road traffic crash victims, helmet use by motorcyclists 24.7%, seat belt use by car occupants 13.5%, alcohol use prior to crash 17.2% |
| Abegaz, T., Berhane, Y., Worku, A., Assrat, A., & Assefa, A. (2014). Effects of excessive speeding and falling asleep while driving on crash injury severity in Ethiopia: A generalized ordered logit model analysis. *Accident Analysis and Prevention 71*, 15-21. | Ethiopia | Addis Ababa-Hawassa highway | police data | 2012-2013 | 819 road crashes | all road users | Alcohol use 9,8%, speeding 52,6% of total injuries, not using seatbelt 20,6%; distribution of types of vehicles involved in accidents |
| Damsere-Derry, J. Ebel, B. E., Mock, C. N., Afukaar, F., & Donkor, P. (2010). Pedestrians’ injury patterns in Ghana. *Accident Analysis and Prevention 42*, 1080–1088. | Ghana | Kumasi–Accra highway | police data | 2002-2006 | 812 fatal & nonfatal RTIs | pedestrians | 27,9% of pedestrian total injuries speeding is a factor; probability that a pedestrian fatality occurring in Ghana attributable to excessive speeding is 65% |
| A profile of fatal injuries in South Africa - 7th Annual Report of the NATIONAL INJURY MORTALITY SURVEILLANCE SYSTEM 2005 | South Africa |  | mortuary data | 2005 | 5675 transport deaths | all road users | Pedestrian & vehicle driver & passenger deaths by age. Car drivers 53,5% BAC positive, passengers 39,7% BAC positive, pedestrians 58,7% BAC positive, cyclists 44,9% BAC positive. |
| TRANSPORT STATISTICS: 2007. STATS BRIEF Released by the Central Statistics Office. Republic of Botswana | Bots­wana |  | police data/official statistics | 2007 | 37463 casualties | all road users | Deaths: alcohol/drugs 15 (3%)  Injuries: alcohol/drugs 201 (2,8%) |

#### 2.2.2 Distribution of risk factors by road users in South-East Asia

Some data on risk factor distribution by road users were found for 4 countries from South-East Asia, a total of 7 articles (Table 5). For India, one comprehensive report for 2011 outlines deaths attributable to intake of alcohol and speeding; other studies are limited to certain road user groups (e.g. two-wheelers).

**Table 5.** Data sources for distribution of risk factors by road users in South-East Asia

| **Reference** | **Country** | **Region** | **Type of data** | **Data period** | **n** | **Road user groups reported** | **Findings** |
| --- | --- | --- | --- | --- | --- | --- | --- |
| Road accidents in India 2011. Government of India, Ministry of Road Transport and Highways, Transport research wing, New Delhi. | India |  | police data | 2011 | 497 686 accidents, 653 879 victims | all road users | accidents caused due to intake of alcohol/drugs 6,4% of accidents, 10,3% of deaths; speeding 59% of accidents, 58,4% of deaths |
| Kakeri, S. R., Bagali, M. A., Goudar, E.S., & Qadri, S. Y. (2014). Pattern of injuries and death sustained by the occupants of the two-wheeler during road traffic accidents. *Al Ameen Journal of Medical Science 7*(2) , 118-124. | India | Bijapur | hospital-based study | 2005-2007 | 150 RTA victims | two-wheelers | 74% two-wheeler road traffic accident victims did not wear helmets |
| Fitzharris, M., Dandona, R., Kumar, R., & Dandona, L. (2009). Crash characteristics and patterns of injury among hospitalizedmotorised two-wheeled vehicle users in urban India. *BMC Public Health 9(*11). | India | Hyderabad city, urban | multiple hospital study | 2005-2006 | 378 motorized two-wheeler users | motorized two-wheelers | 19.6% of injured and deaths wore a helmet correctly; 80,4% of injured and deaths did not wear a helmet |
| Waseela M, & Laosee O. (2014). Determinants of Road Traffic Injury Among Adult Motorcyclists in Malé, Maldives. A*sian Pacific Journal of Public Health. 2014 Jun 23*. [Epub ahead of print] | Maldives | Malé | survey data | 2012-2013 | 294 motorcycle riders | motorcyclists | Excessive speed 14,5% as the primary cause for motorcycle RTIs |
| Weerawardena, W. A. K., Illanagasingha, T. D. B, Piyadasa, I. J., Rathnayaka, S.M., Subaweera, W.T.D.U.P.L., & Niroshana, G.A.L. (2013). Analysis of patients admitted with history of Road Traffic Accidents to surgical unit B Teaching Hospital Anuradhapura, Sri Lanka. *Anuradhapura Medical Journal 7*(1), 2-5. | Sri Lanka | Anuradhapura | hospital-based study | 2012-2013 | 214 patients | all road users, admitted patients | distribution of injuries by vehicle types & road users, 32% alcohol use, 39% not wearing a helmet. |
| Nakahara, S., Chadbunchachai, W., Ichikawa, M., Tipsuntornsak, N., & Wakai, S. (2005). Temporal distribution of motorcyclist injuries and risk of fatalities in relation to age, helmet use, and riding while intoxicated in Khon Kaen, Thailand. *Accident Analysis and Prevention 37*, 833–842. | Thailand | Khon Khaen | hospital-based study | 1998-2002 | 9948 injured motorcyclists | motorcyclists | fatal & nonfatal cases of motorcyclist injuries, 74,9% not wearing a helmet, 36,5% drink-driving. |

### 2.3. Fatal and non-fatal injuries by road users

#### 2.3.1 Fatal and non-fatal injuries by road users, Sub-Saharan Africa

Report by WHO, “Global Status Report on Road Safety 2013” provides data for fatal road traffic injures by country and road user type. Other than that, data for multiple Sub-Saharan Africa countries were found (a total of 11 data sources). Also some regional data were reported on WHO factsheets, and a review from 2009 by Naci, Chisholm & Baker. Usually most RTI studies provide some distribution by road users, although the categories may not correspond to those used previously, and an age distribution of these road users is usually missing. Table 6 shows data sources for RTI distribution by road users in Sub-Saharan Africa region. Most studies find pedestrians the most vulnerable road user group, accounting for 19-60% of RTIs, followed by car occupants and motorcycle riders. The share of car occupants in RTI casualties in Sub-Saharan Africa has increased when compared to previous analysis.

**Table 6.** Data sources for fatal and non-fatal injuries by road users, Sub-Saharan Africa

| **Reference** | **Country** | **Region** | **Type of data** | **Data period** | **n** | **Road user groups / fatality** | **Findings** |
| --- | --- | --- | --- | --- | --- | --- | --- |
| ROAD SAFETY IN THE WHO AFRICAN REGION. THE FACTS 2013 (WHO factsheet) | African region | - | WHO data | 2013 | - | all road users, fatal injuries | Deaths: 43% vehicle occupants, 38% pedestrians, 7% cyclists, 7% 2- & 3-wheeler occupants, 5% other |
| Naci, H., Chisholm, D., Baker, T. D. (2009). Distribution of road traffic deaths by road user group: a global comparison. *Injury Prevention 15,* 55–59 | African region, South-East Asia Region | - | literature review | 1991-2006 | - | all road users, fatal injuries | Deaths: AFRO-E motorized four-wheelers 29%, motorcyclists 5%, bicyclists 11%, pedestrians 55%; SEAR-D motorized four-wheelers 19%, motorcyclists 43%, bicyclists 8%, pedestrians 30% |
| Macharia, W. M., Njeru, E. K., Muli-Musiime, F., & Nantulya, V. (2009). Severe road traffic injuries in Kenya, quality of care and access. *African Health Sciences 9*(2), 118-124. | Kenya | - | hospital-based/survey study (sample: 50 hospitals) | 1997-1998 | 310 RTI casualties | all road users, nonfatal injuries | **Of RTI victims:** owner 2%, employee driver 4,2%, passenger 47,2%, pedestrian 32,9%, unspecified 13,7%. **Of crash vehicles:** private cars 20,3%, buses 30%, commuter mini-buses 43,6%, lorries 12,9%, pedal/motorcycles 3,9%, unspecified 9,5% |
| Ogendi, J., Odero, W., Mitullah, W., & Khayesi, M. (2013). Pattern of pedestrian injuries in the city of Nairobi: implications for urban safety planning. *Journal of Urban Health 90*(5). | Kenya | City of Nairobi | hospital-based study | 2011 | 176 persons with RTIs | all road users, fatality not known | Pedestrians comprised the highest (59.1 %) proportion of road traffic injury admissions, followed by motor vehicle passengers (24.4 %) and motor cyclists (9.7 %). Bicyclists and drivers accounted for 5.1 and 1.7 %, respectively |
| Damsere-Derry, J. Ebel, B. E., Mock, C. N., Afukaar, F., & Donkor, P. (2010). Pedestrians’ injury patterns in Ghana. *Accident Analysis and Prevention 42*, 1080–1088. | Ghana | - | surveillance/police data | 2002-2006 | 812 pedestrian casualties | pedestrians | distribution of fatal & non-fatal injuries by vehicle type, sex and injury severity / bus occupants 15,4% non-fatal, 11,0% fatal; motorcycle 2,9% non-fatal, 0,4% fatal; bicycle 0,5% non-fatal, 0% fatal |
| Whiteside, L.K., Oteng, R., Carter, P., Amuasi, J., Abban, E., Rominski, S., Nypaver, M., & Cunningham, R.M. (2012) Non-fatal injuries among pediatric patients seeking care in an urban Ghanaian emergency department. *International Journal Of Emergency Medicine 5* (1), 36. | Ghana | Kumasi | hospital-based study | 2009 | 50 RTI patients | children only | pediatric road traffic injuries by road user type: 58% (29) car crash, 26% (13) pedestrian injury, 14% (7), bicycle crash 2%(1) |
| <http://www.arrivealive.co.za/documents/FATAL%20CRASHES%20PER%20MONTH_Nov_2011-March_2012.pdf> | South Africa | All provinces | surveillance data | 2011-2012 | 5514 fatalities | drivers, passengers, pedestrians | Drivers (1681) 30,5%, passengers (1890) 34,3%, pedestrians (1944) 35,3% |
| Abegaz, T., Berhane, Y., Worku, A., & Assrat, A. (2014). Effectiveness of an improved road safety policy in Ethiopia: an interrupted time series study. *BMC Public Health 14*(539). | Ethiopia | Addis Ababa - Adama/Hawassa main road | police data/crash records | 2002-2011 | 4,053 crashes, of those 1193 fatal & 980 non-fatal injury crashes (1392 fatalities, 1749 injuries) | all road users | From 1,193 fatal crashes 1,392 people were dying, on average 1.2 deaths per fatal crashes. Of these deaths, more than half 7.5% (800) were pedestrian, 32% (445) vehicle occupants and 10.5% (147) drivers. During the 980 injury crashes 1,749 people were injured, on average, 1.8 injuries per crash, over half, 55.2% (965) were vehicle occupants, followed by pedestrian 35.1% (614) and the rest 9.7% (170) were drivers. |
| TRANSPORT STATISTICS: 2007. STATS BRIEF Released by the Central Statistics Office. Republic of Botswana | Botswana |  | police data/official statistics | 2003-2007 | 37463 casualties | all road users | Casualities by road user: 19,6% pedestrians, 1,1% cyclists, 1,1% motorcyclists, 30% car occupants (excl. taxi, 4WD, pickup), 24,7% pickup user, 8,1% bus or minibus user. |
| Chalya, P. L., Mabula, J. B., Dass, R. M., Mbelenge, N., Ngayomela, I. H., Chandika, A. B., & Gilyoma, J. M. (2012). Injury characteristics and outcome of road traffic crash victims at Bugando Medical Centre in Northwestern Tanzania. *Journal of Trauma Management & Outcomes 6*(1). | Tanzania | Northwestern Tanzania | hospital-based study | 2010-2011 | 1678 road traffic crash victims | all road users | Motorcycle (986, 58.8%) was responsible for the majority of road traffic crashes, followed by motor-vehicles (650, 38.7%), bicycle (36, 2.1%) and other means of transport (e.g. donkey, trolley etc) in 4 (0.2%) of cases. Pedestrians (930, 55.4%) accounted for the majority of victims, followed by passengers (457, 27.2%), drivers/riders (287, 17.2%) and others (4, 0.2%). |
| Masaoe, E.N. (2007). Study on Road Accidents in Mainland Tanzania. Final Report submitted to Surface and Marine Transport Regulatory Authority (SUMATRA). | Tanzania |  | police data | 2000-2005 | 12538 fatalities & 92123 injured in 2000-2005 | all road users | fatal & non-fatal road traffic injuries by road user type & vehicle type - **fatal:** car occupants (drivers 11,6% + passengers 42,8%) 54,4%; motorcyclists 3,9%; pedal cyclists 8,9%; pedestrians 32,9%. **Non**-**fatal:** car occupants 59,6%; motorcyclists 2,8%; pedal cyclists 7,0%; pedestrians 30,6%. |
| Komba, D. D. (2006). *Risk Factors and Road Traffic Accidents in Tanzania: A Case Study of Kibaha District.* Master Thesis in Development Studies, Specialising In Geography Department of Geography Norwegian University of Science and Technology (NTNU). | Tanzania | Kibaha / Tumbi hospital | hospital data | 2001-2004 | 764 | Age groups 0-17, 18-24, 25-34, 35-44, 45+ | age distribution of injuries by road user type: car occupants 75,8%, pedestrians 21%, motorcyclists 1,7% & cyclists 1,6% of all injured persons |

#### 2.3.2 Fatal and non-fatal injuries by road users, South-East Asia

In addition to already aggregated regional data for South-East Asia (Naci et al. 2009; Road Safety Status in the… 2013), data were found for 4 South-East Asia countries: India, Sri Lanka, Vietnam and Malawi (a total of 13 articles) (Table 7). Numerous data sources were found for India (although mainly small hospital-based and autopsy studies). The share of motorcyclists’ and other motorized light vehicle occupants among all RTI deaths and injured is typically high; although in some regions pedestrians appear to be the most endangered road user group (Malawi, Northern India). Compared to previous report the share of motorized light vehicles appears to be even higher during recent years.

**Table 7.** Data sources for datal and non-fatal injuries by road users in South-East Asia

| **Reference** | **Country** | **Region** | **Type of data** | **Data period** | **n** | **Road user groups / fatality** | **Findings** |
| --- | --- | --- | --- | --- | --- | --- | --- |
| Naci, H., Chisholm, D., Baker, T. D. (2009). Distribution of road traffic deaths by road user group: a global comparison. *Injury Prevention 15,* 55–59 | South-East Asia Region | - | literature review | 1991-2006 | - | all road users, fatal injuries | Deaths: SEAR-D motorized four-wheelers 19%, motorcyclists 43%, bicyclists 8%, pedestrians 30% |
| Road safety status in the WHO South-East Asia Region, 2013 (WHO factsheet) | South-East Asia Region | - | WHO data | 2009-2010 | - | all road users, fatal injuries | Deaths: 33% motorized two- or three-wheelers, 12% pedestrians, 4% cyclists, 15% car occupants, 36% unspecified |
| Hsiao, M., Malhotra, A., Thakur, J.S., et al. (2013). Road traffic injury mortality and its mechanisms in India: nationally representative mortality survey of 1.1 million homes. *BMJ Open 2013(3*):e002621. | India | - | large mortality survey, verbal autopsy | 2001-2003 | 2299 RTI deaths in the survey correspond to an estimated 183 600 RTI deaths or about 2% of all deaths in 2005 nationally | all road users | estimated road traffic deaths by road user type: pedestrians 37%, motorcyclists 20%, car occupants 16%, bicyclists 8% (+three-wheelers 3%). |
| Road accidents in India 2011. Government of India, Ministry of Road Transport and Highways, Transport Research Wing, New Delhi 2012. I | India | - | official statistics | 2011 | 142 485 killed; 511 394 injured (in 497686 accidents) | all road users | **% of killed:** 19,2% two-wheelers; 17,6% cars, 36,6% buses/trucks; **of injured:** 22,5% two-wheelers; 20,4% cars, 32,6 buses/trucks |
| Patil, S. S., Kakade, R. V., Durgawale, P. M., & Kakade, S. V. (2008). Pattern of Road Traffic Injuries: A Study from Western Maharashtra. Indian Journal of Community Medicine 33(1), 56-57. | India | Western Maharashtra | hospital-based study | 2003-2004 | 350 RTIs | all road users | road traffic injuries by road user type, drivers & passengers separately reported. Of casualities: 82,3% male, 17,7% female, highest number (29,4%) between 20-29 years of age. Pedestrians 13,4%, bicyclists 21,7% of drivers. |
| Manish, K, Jyothi, N. S, Pawar, G. S., Jatti, V. B. (2012). Fatal Head Injuries in Road Traffic Accidents in and around Davangere: A Prospective Study. Indian Journal of Forensic Medicine and Pathology 5(2). | India | Davangere | hospital-based study | 2005-2007 | 408 RTI deaths | all road users | of fatalities: 46,3% motorcyclists, 31,7% pedestrians, 4,8% cyclists, 3,6% car occupants, 4,8% bus or truck occupants |
| Das, R. K., Chakraborty, P. N., Das, P. (2014). A Study of the pattern of Cranio-Facial Injuries in Fatal Road Traffic Accidents in Tripura. *Journal of Evolution of Medical and Dental Sciences 3*(24), 6726-6735. | India | Tripura | autopsy study | 2011-2013 | 196 victims of fatal RTIs, craniofacial injuries | RTI victims with cranio-facial injuries | fatal RTI victims w/ craniofacial injuries: pedestrians 42,9%; car occupants 16,3; bicyclists 5,1%. |
| Khajuria, B., Sharma, R., Verma, A. (2008). A Profile of the Autopsies of Road Traffic Accident Victims in Jammu. *Journal of Clinical and Diagnostic Research 2*, 639-642. | India | Jammu | autopsy study | 2000-2005 | 249 RTA victims | all road users | fatal RTI victims: pedestrians 55,32%, vehicle occupants 34,68 |
| Sharma, B. R., Sharma, A. K., Sharma, S. & Singh, H. (2007). Fatal Road Traffic Injuries in Northern India: Can They Be Prevented? *Trends in Medical Research 2*(3), 142-148. | India | Northern India | hospital-based study, autopsies | 1996-2005 | 1109 RTA victims | all road users | Fatal RTIs: pedestrians 38,7%, motorized two-wheelers 34,1%, cyclists 5,9%, light motor vehicles 10,4%, bus occupants 2,9%. |
| Jain, A., Menezes, R. G., Kanchan, T., Gagan, S., & Jain, R. (2009). Two wheeler accidents on Indian roads – a study from Mangalore, India. *Journal of Forensic and Legal Medicine 16*, 130–133. | India | Mangalore | police data | 2000-2004 | 1231 two-wheeler accidents | two-wheelers only | age distribution of two-wheeler accident victims / age groups <18, 18-24, 25-34, 35-44, 45-54, >55 |
| *Road Safety in India: A Framework for Action.* (2011). National Institute of Mental Health & Neuro Sciences, WHO Collaborating Centre for Injury Prevention & Safety Promotion, Department of Epidemiology. | India | - | official statistics | 2009-2010 | 160000 estimated RTI deaths | all road users | approximate distribution of fatal & non-fatal road crashes by urban/rural & by road user type. Fatal: ~40% pedestrians, 40% two-wheeler riders & pillions. Non-fatal: 25% pedestrians, 50% two-wheeler riders & pillions |
| Honnungar, R. S., Aramani, S. C., Vijay Kumar, A. G., Ajay Kumar, T. S, Jirli, P. S. (2011). An Epidemiological Survey of Fatal Road Traffic Accidents and their Relationship with Head Injuries. *Journal of Indian Academic Forensic Medicine 33*(2), 135-137. | India | Karnataka | autopsy study | 2004-2009 | 506 vehicle accident fatalities | all road users | Fatal injuries: pedestrians 16,4%, pedal-cyclists 13,1%, motorcyclists 28,3%, drivers 42,2% |
| Somasundaraswaran, A. K. (2006). Accident Statistics in Sri Lanka. *IATSS Research 30*(1). | Sri Lanka | - | police data | 1989-2005 | All reported accidents during the period (43171 in 2005) | all road users | fatalities & casualties by road user type. Fatalities in 2005: 32,4% pedestrians, 26,3% car occupants, 14,1% bicyclists. Non-fatal in 2001: 30,8% pedestrians, 12,2% bicyclists, car occupants 40,7% |
| Weerawardena, W. A. K, Illanagasingha, T. D. B., Piyadasa, I.J., Rathnayaka, S. M., Subaweera, W. T. D. U. P. L., Niroshana, G. A. L. (2013). Analysis of patients admitted with history of Road Traffic Accidents to surgical unit B Teaching Hospital Anuradhapura, Sri Lanka. *Anuradhapura Medical Journal 7*(1), 2-5. | Sri Lanka | Anuradhapura | hospital-based study | 2012-2013 | 214 RTI patients | all road users | fatal & nonfatal injuries. Vehicle type involved with the injury: motorcycle 138(65%), bicycles 23(11%), three wheelers 23(11%), tractors 11(5%), buses 5(2%), lorries 6(3%), cars 2(1%) and other 3(1%). There were 135(64%) drivers/riders, 59(28%) passengers and 17(8%)pedestrians. |
| Ngo, A.D., Rao, C., Phuong Hoa, N., Hoy, D. G., Quynh Trang, K. T., & Hill, P. S. (2012). Road traffic related mortality in Vietnam: Evidence for policy from a national sample mortality surveillance system. *BMC Public Health 12*, 561. | Vietnam |  | statistics from surveillance system | 2008-2009 | 1,061 deaths attributable to road crashes | all road users | Of deaths: 11,2% pedestrians, 3,2% cyclists, 57,9% motorcyclists, 2,45% car occupants |
| Samuel, J. C., Sankhulani, E., Qureshi, J. S., Baloyi, P., Thupi, C., et al. (2012). Under-Reporting of Road Traffic Mortality in Developing Countries: Application of a Capture-Recapture Statistical Model to Refine Mortality Estimates. *PLoS ONE 7*(2): e31091. | Malawi |  | hospital data, police data, estimated number | 2008-2009 | 380 estimated RTI deaths | all road users | road traffic deaths: 42,4% pedestrians, 10,3% bicyclists, 36,3% car occupants. |

## 3. Road safety interventions

Search keywords used were country name+road safety, country name+speed bumps, country name+drink-driving, country name+speed cameras, country name+helmet use.

No data were found on coverage of speed humps/bumps, drink-driving law & enforcement nor coverage of speed cameras in Sub-Saharan Africa or South-East Asia countries.

Some data were found on seat belt, motorcycle and bicycle helmet use. Road safety intervention statistics are based mainly on surveillance data, and some survey data.

### 3.1. Seat belts, Sub-Saharan Africa

The percentage of vehicle occupants wearing a seatbelt was only available for South Africa (Table 8).

**Table 8.** Data sources for seat belt use in Sub-Saharan Africa

| **Reference** | **Country** | **Region** | **Type of data** | **Data period** | **n** | **Road user groups reported** | **Findings** |
| --- | --- | --- | --- | --- | --- | --- | --- |
| van Hoving, D. J., Sinclair, M., Wallis, L. A. (2013). Patterns of seatbelt use in different socioeconomic communities in the Cape Town Metropole, South Africa. *African Medical Journal 103*(9). | South Africa | Cape Town Metropole | surveillance data | 2010 | 4 651 vehicles with 6 848 occupants were surveyed | vehicle occupants | vehicle occupants, 45.1% wearing a seatbelt |

### 3.2 Seat belts, South-East Asia

WHO factsheets on South-East Asia Region provide numbers for seat belt use in India & Sri Lanka, also two studies for India report some estimates for seat belt use among car occupants (Table 9). The estimates differ, although the study reporting the highest percentages of seat belt use (Mohan, 2009) is the only one that describes the methods how the estimates were calculated.

**Table 9.** Data on seat belt use in South-East Asia

| **Reference** | **Country** | **Region** | **Type of data** | **Data period** | **n** | **Road user groups reported** | **Findings** |
| --- | --- | --- | --- | --- | --- | --- | --- |
| Road safety status in the WHO South-East Asia Region, 2013 (WHO factsheet) | India, Sri Lanka |  | unknown | n/a | n/a |  | seat belt use: 27% in India; 79% in Sri Lanka |
| Mohan, D. (2009). Seat Belt Law and Road Traffic Injuries in Delhi, India. Proceedings of the Eastern Asia Society for Transportation Studies 7. | India | urban | surveillance data | 2002-2005 | 5,315 cars, average of 2.2 persons per car | vehicle occupants | Front seat passengers: male drivers – 82%; female drivers – 80%; male passengers – 58%; female passengers – 61% |
| Gururaj G. (2011). Road safety in India: a framework for action. National Institute of Mental Health and Neuro Sciences, Publication no 83, 1–40. | India | Banga­lore city, urban | official statistics? | n/a | n/a | car drivers | only 27% of car drivers wear seat belts |

### 3.3. Motorcycle helmet use, Sub-Saharan Africa

WHO factsheet on African Region provides numbers for motorcycle helmet wearing rates for Congo, South Africa, Seychelles, Botswana. Data from other sources: some motorcycle helmet use statistics were available for Kenya (Table 10).

**Table 10.** Data on motorcycle helmet use in Sub-Saharan Africa

| **Reference** | **Country** | **Region** | **Type of data** | **Data period** | **n** | **Road user groups reported** | **Findings** |
| --- | --- | --- | --- | --- | --- | --- | --- |
| ROAD SAFETY IN THE WHO AFRICAN REGION. THE FACTS 2013 (WHO factsheet) | - | - | official statistics | 2013? | n/a | motorcyclists | motorcycle helmet wearing rates for: 3% in Congo to 95% in both South Africa and Seychelles, and 100% in Botswana. |
| Bachani, A. M., Koradia, P., Herbert, H. K., Mogere, S., Akungah, D., Nyamari, J., Osoro, E., Maina, W., & Stevens, K. A. (2012) Road Traffic Injuries in Kenya: The Health Burden and Risk Factors in Two Districts, *Traffic Injury Prevention, 13*(sup1), 24-30. | Kenya | Thika, Naivasha | surveillance data | 2010 | 3075 (Thika), 3143 (Naivasha) | motorcyclists | Thika: 30,37% of drivers, 4,06% of passengers / Naivasha 21,29% of drivers, 2,61% of passengers |

### 3.4. Motorcycle helmet use, South-East Asia

Motorcycle helmet use in South-East Asia is better documented in Vietnam, where after compulsory helmet use legislation and enforcement in 2007 helmet wearing rates increased from 27% to 99% in drivers; and 21% to 99% in passengers. For India we have two self-reported estimates from surveys, the more clear one marks motorcycle helmet use at 64% in India (Table 11).

**Table 11.** Data on motorcycle helmet use in South-East Asia

| **Reference** | **Country** | **Region** | **Type of data** | **Data period** | **n** | **Road user groups reported** | **Findings** |
| --- | --- | --- | --- | --- | --- | --- | --- |
| Mirkazemi, R., Kar, A. (2009). Injury-related unsafe behavior among households from different socioeconomic strata in Pune city. Indian Journal of Community Medicine 34(4), 301-305. | India | Pune city | survey data | 2007-2008 | 200 house­holds | two-wheeled vehicle riders | Two-wheeled vehicle riders: 35,6% did not have a helmet and 57,7% of those who had one, did not use it regularly |
| Gururaj G. (2011). Bangalore road safety and injury prevention program - results and learning 2007-2010. National Institute of Mental Health and Neuro Sciences. Publication No 81 | India | Bangalore | survey data | 2011 | 145789 two wheeler riders | motorcyclists | The use of helmets was only 64%. 49% of urban and 80% of rural injured motorcyclists had not worn helmets at the time of crash. |
| Passmore, J. W., Nguyen, L. H., Nguyen, N. P., & Olivé, J-M. (2010). The formulation and implementation of a national helmet law: a case study from Viet Nam. Bulletin of the World Health Organization 88(10), 783-787. | Vietnam |  | surveillance data | 2007-2008 | n/a | motorcyclists | approximate % of motorcycle riders wearing a helmet before and after legislation on compulsory helmet use. In Da Nang, helmet wearing increased from 27% to 99% in drivers; and 21% to 99% in passengers |

### 3.5. Bicycle helmet use, South-East Asia

Bicycle helmet use was documented in one study from Singapore, and this study only evaluated the helmet use of bicycle-related trauma patients, and placed the estimate at 10,6% (Table 12).

**Table 12.** Data on bicycle helmet use, South-East Asia

| **Reference** | **Country** | **Region** | **Type of data** | **Data period** | **n** | **Road user groups reported** | **Findings** |
| --- | --- | --- | --- | --- | --- | --- | --- |
| Heng, K. W., Lee, A. H., Zhu, S., Tham, K. Y., & Seow, E. (2006). Helmet use and bicycle-related trauma in patients presenting to an acute hospital in Singapore. *Singapore Medical Journal 47*(5), 367-372. | Singapore | - | survey data | 2004-2005 | 160 bicyclists | bicyclists, trauma patients | % of bicycle-related trauma patients wearing a helmet: helmets were worn by 10.6 percent of the patients |

## 4. Sequelae of road traffic accidents

A standardized online keyword search was carried out to obtain country specific risk factor information, using online search engines such as Google, Google Scholar, and EBSCO. We tried to find information on sequelae by injury categories used in the previous report: fractured skull, intracranial injuries, fractured femur, injured spinal chord, injury to eyes. Keywords used were country name+road traffic+skull fracture, country name+road traffic+intracranial injuries, country name+road traffic+femur fracture, country name+road traffic+spine injury, country name+road traffic+eye injury

No studies provided the exact distribution of sequelae used in the previous report (fractured skull, intracranial injuries, fractured femur, injured spinal cord and injury to eyes). Some studies differentiated between soft-tissue injuries and fractures, some divided injuries into categories by body part injured. If the study distinguished between fatal & non-fatal injuries, the distinction between long-term and acute injuries was impossible to make.

### 4.1 Sequelae of road traffic accidents, Sub-Saharan Africa

Some hospital-based and survey data for Tanzania and Kenya were found. There was a lot of variation in estimates for fractured skull as a percentage from all road traffic injuries. Some studies did not differentiate between different head injuries. The results of the studies seem to indicate that head injury is present in at least 10% of fatal and non-fatal RTIs, but more likely is the rate of 25% and higher (Table 13).

**Table 13.** Data on sequelae of road traffic accidents, Sub-Saharan Africa

| **Reference** | **Country** | **Region** | **Type of data** | **Data period** | **n** | **Road user groups reported** | **Findings** |
| --- | --- | --- | --- | --- | --- | --- | --- |
| Akama, M. K., Chindia, M. L., Macigo, F. G., & Guthua, S. W. (2007). Pattern of maxillofacial and associated injuries in road traffic accidents. *East African Medical Journal 84*(6, 287-95. | Kenya | Nairobi | hospital-based study | n/a | 482 | all road users | skull fractures, % of all fatal & non-fatal injuries / head injury 37,7%, ~5% skull fractures |
| Gichuhi , K. (2007). Injury Pattern Among Non-fatal Road Traffic Crash Victims. *East African Orthopaedic Journal 1*. 23-25. | Kenya | Nairobi | hospital-based study | 2004 | 1424 victims of road traffic crashes | all road users | head injury 25,6%, femoral fracture 12,4%, spine injury 1,1%, ruptured eye 0,2%, % of all fatal & non-fatal injuries |
| Masaoe, E. N. (2007). Study on Road Accidents in Mainland Tanzania. Final Report submitted to Surface and Marine Transport Regulatory Authority (SUMATRA). http://www.sumatra.or.tz/index.php/component/docman/doc_view/49-study-on-road-accidents-in-mainland-tanzania?Itemid=317 (Accessed on 24.07.2014) | Tanzania |  | survey of RTI survivors & relatives | 1994-2007 | 102 RTI victims/relatives | all road users | Head injuries 11%, back injuries 10%, sight problems 2% of all fatal & non-fatal injuries |
| Chalya, P. L., Mabula, J. B., Dass, R. M., Mbelenge, N., Ngayomela, I. H., Chandika, A. B., & Gilyoma, J. M. (2012). Injury characteristics and outcome of road traffic crash victims at Bugando Medical Centre in Northwestern Tanzania. *Journal of Trauma Management & Outcomes 6*(1). | Tanzania | Northwestern Tanzania | hospital-based survey | 2010-2011 | 1678 road traffic crash victims | all road users | all head injuries 52.1%, spinal fractures 1,4%, skull/maxillofacial fractures 19,7%, pelvic fractures 3,6%, % of all fatal & non-fatal injuries |

### 4.2 Sequelae of road traffic accidents, South-East Asia

As for South-East Asia region, only available data on sequelae of RTAs originated from India (8 studies, 5 of them autopsy studies, the rest hospital-based or surveillance data, see Table 14). The data seem to indicate that head injuries, including skull fracture, are present at up to 70% of fatal RTIs; the prevalence of the rest of the sequelae vary depending on the road user status of accident victims and other circumstances of the injury.

**Table 14.** Data on sequelae of road traffic accidents, South-East Asia

| **Reference** | **Country** | **Region** | **Type of data** | **Data period** | **n** | **Road user groups reported** | **Findings** |
| --- | --- | --- | --- | --- | --- | --- | --- |
| Manish, K, Jyothi, N. S, Pawar, G. S., Jatti, V. B. (2012). Fatal Head Injuries in Road Traffic Accidents in and around Davangere: A Prospective Study. Indian Journal of Forensic Medicine and Pathology 5(2). | India | Davangere | autopsy study | 2005-2007 | 408 RTI deaths | all road users | fractured skull, 40,1% of all fatal injuries |
| Sharma, B.R., Sharma, A.K., Sharma, S. & Singh, H. (2007). Fatal Road Traffic Injuries in Northern India: Can They Be Prevented? *Trends in Medical Research 2*(3), 142-148. | India | Northern India | autopsy study | 1996-2005 | 1109 autopsies | all road users | head injury w/ skull fracture 52,4%, head injury w/o skull fracture 8,6%, % of all fatal injuries |
| Patil, S. S., Kakade, R. V., Durgawale, P. M., & Kakade, S. V. (2008). Pattern of Road Traffic Injuries: A Study from Western Maharashtra. Indian Journal of Community Medicine 33(1), 56-57. | India | Western Maharashtra | hospital-based study | 2003-2004 | 350 RTIs | all road users | 13,2% skull fracture |
| Kumar, A., Lalwani, S., Deepak, A., Rautji. R., & Dogra, T. D. (2008). Fatal road traffic accidents and their relationship with head injuries: An epidemiological survey of five years. *Indian Journal of Neurotrauma 5*(2), 63-67. | India |  | autopsy study | 2001-2005 | 2472 autopsies of vehicular accidents | all road users | 68,7 head injury, 69,6% skull fracture, intracranial hemorrhage ~89%, spine fracture 6,4%; % of all fatal injuries |
| Khajuria, B., Sharma, R., & Verma, A. (2008). A profile of the autopsies of road traffic accident victims in Jammu. *Journal of Clinical and Diagnostic Research 2*, 639-642 | India |  | autopsy study | 2000-2005 | 249 RTA victims | all road users | of deaths: head injury 69,48%; spine injury 0,8% / of injuries: head injury 28,62%; spine injury 0,82% |
| Honnungar, R. S., Aramani, S. C., Vijay Kumar, A. G., Ajay Kumar, T. S, Jirli, P. S. (2011). An Epidemiological Survey of Fatal Road Traffic Accidents and their Relationship with Head Injuries. *Journal of Indian Academic Forensic Medicine 33*(2), 135-137. | India | Karnataka | autopsy study | 2005-2009 | 506 vehicle accident fatalities | all road users, medico legal cases autopsied | skull fracture 77,7%; subdural hemorrhage 73,9%; % of all fatal injuries |
| Fitzharris, M., Dandona, R., Kumar, R., & Dandona, L. (2009). Crash characteristics and patterns of injury among hospitalized motorised two-wheeled vehicle users in urban India. *BMC Public Health 9*(11). | India | Hyderabad, Urban | hospital-based study | 2005-2006 | 378 | motorized two-wheelers only | head fracture 10,3%; intracranial injuries 11,1-11,5%; % of all non-fatal & fatal injuries in two-wheeler riders & pillions |
| *Bengaluru Injury / Road Traffic Injury Surveillance Programme: A feasibility study.* (2008). National Institute of Mental Health & Neuro Sciences | India | Bengaluru | surveillance data / official statistics | 2001 | 2542 fatal and 48775 non-fatal injuries | car occupants only | fatal: head 77%, spine 5% / non-fatal: head 43%, spine 2% |
