# Supplementary material for: Cost-effectiveness of strategies to prevent road traffic injuries in eastern sub-Saharan Africa and Southeast Asia: new results from WHO-CHOICE
Source: Cost Eff Resour Alloc. 2018 Nov 20;16:59. doi: 10.1186/s12962-018-0161-4 (PMC6245850; doi:10.1186/s12962-018-0161-4)
Supplement: Supplementary file 2 — Additional file 2. Effect sizes and costing assumptions. [file 12962_2018_161_MOESM2_ESM.docx]

# Cost-effectiveness of strategies to prevent road traffic injuries in eastern sub-Saharan Africa and Southeast Asia: new results from WHO-CHOICE

# Additional file 2: Effect sizes and costing assumptions

Table 1: Intervention effect sizes used in the analysis

| **Intervention** | **Effect on RTI** | **Size of effect (by type of road user)** | | | | | |
| --- | --- | --- | --- | --- | --- | --- | --- |
|  |  | Pedestrian | Bicyclist | Motorcyclist | Cars / vans | Buses | Other |
| Enforcement of speed limits (via mobile speed cameras) | Incidence of L-T RTI (non-fatal) | -6% | -6% | -6% | -6% | -6% | -6% |
|  | Crash mortality rate (fatal) | -14% | -14% | -14% | -14% | -14% | -14% |
| Drink-drive legislation & enforcement (via breath-testing campaigns) | Incidence of L-T RTI (non-fatal) | -15% | -15% | -15% | -15% | -15% | -15% |
|  | Crash mortality rate (fatal) | -25% | -25% | -25% | -25% | -25% | -25% |
| Legislation & enforcement of seat belt use in cars (drivers and passengers) | Incidence of L-T RTI (non-fatal) | - | - | - | -18% | - | - |
|  | Crash mortality rate (fatal) | - | - | - | -11% | - | - |
| Legislation & enforcement of helmet use by motorcyclists (all riders) | Incidence of L-T RTI (non-fatal) | - | - | -18 to -29% | - | - | - |
|  | Crash mortality rate (fatal) | - | - | -36% | - | - | - |
| Legislation & enforcement of helmet use by bicyclists aged below 15 years | Incidence of L-T RTI (non-fatal) | - | -17 to -28% | - | - | - | - |
|  | Crash mortality rate (fatal) | - | -69% | - | - | - | - |

Source: Road traffic injury prevention: an assessment of risk exposure and intervention cost effectiveness in different world region, 2008 [5], Table 2

Table 2: Long-term non-fatal road traffic injury: mortality risk and disability level

|  | **% of incident episodes with long-term effects** | | **% of non-fatal RTI burden (% of long-term burden)*** | | **Relative risk of mortality** | **Disability weight** |
| --- | --- | --- | --- | --- | --- | --- |
|  | **Southeast Asia** | **Eastern sub-Saharan Africa** | **Southeast Asia** | **Eastern sub-Saharan Africa** |  |  |
| Fractured skull | 15% | 15% | 6% (1%) | 5% (2%) |  |  |
| Intracranial injuries | 13% | 5% | 20% (82%) | 16%(64%) |  |  |
| Fractured femur | 5% | 5% | 14% (1%) | 21% (2%) |  |  |
| Injured spinal chord | 100% | 100% | 38% (11%) | 22% (21%) |  |  |
| Injury to eyes | 10% | 10% | 7% (6%) | 13% (10%) |  |  |
| Weighted average(Southeast Asia) |  |  |  |  | 4.0 | 0.524 |
| Weighted average(Eastern sub-Saharan Africa) |  |  |  |  | 4.3 | 0.455 |

Calculated based on data provided by the International Injury Research Unit at the Johns Hopkins University

Table 3: Coverage per intervention

| **Intervention** | **Coverage\Region** | **Southeast Asia** | **Eastern sub-Saharan Africa** |
| --- | --- | --- | --- |
|  |  |  |  |
| Enforcement of speed limits (via mobile speed cameras) | Baseline coverage | 10% | 5% |
|  | Target coverage | 80% | 80% |
| Drink-drive legislation & enforcement (via breath-testing campaigns) | Baseline coverage | 10% | 10% |
|  | Target coverage | 80% | 80% |
| Legislation & enforcement of seat belt use in cars (drivers and passengers) | Baseline coverage | 0% | 0% |
|  | Target coverage | 50% | 50% |
| Legislation & enforcement of helmet use by motorcyclists (all riders) | Baseline coverage | 30% | 30% |
|  | Target coverage | 90% | 90% |
| Legislation & Enforcement of helmet use by bicyclists aged below 15 years | Baseline coverage | 5% | 5% |
|  | Target coverage | 80% | 80% |

Table 4: Traffic law enforcement costing assumptions

| **Variable\Interventions** | **Speed cameras** | **Breath-testing (alcohol)** | **Seat belts** | **Motorcycle helmets** | **Bicycle helmets** |
| --- | --- | --- | --- | --- | --- |
| % vehicles pulled over per annum | 10% | 10% | 10% | 20% | 5% |
| Vehicles processed per officer per hour | 4 | 4 | 4 | 4 | 4 |
| Officers per checkpoint | 3 | 3 | 2 | 3 | 2 |
| Duration of checkpoint (hours) | 4 | 4 | 4 | 4 | 2 |
| Set-up / dismantle / paperwork time (hours) | 2 | 2 | 2 | 2 | 1 |
| Vehicles used per checkpoint | 2 | 2 | 1 | 0 | 0 |
| Traffic cones used per checkpoint (sets of 10) | 2 | 2 | 2 | 0 | 0 |
| Breathalyser kits used per checkpoint | 0 | 1 | 0 | 0 | 0 |
| Speed cameras used per checkpoint | 1 | 0 | 0 | 0 | 0 |

Table 5: Number of vehicles per 1000 population

| Per 1000 population | Southeast Asia | Eastern sub-Saharan Africa |
| --- | --- | --- |
| Vehicle rate – cars | 28 | 29 |
| Vehicle rate - motorcycles | 309 | 4 |
| Vehicle rate - bicycles | 127 | 43 |

Table 6: Seatbelt and helmet purchase costs

| **Variable** | **Seat belts** | **Motorcycle helmets** | **Bicycle helmets** |
| --- | --- | --- | --- |
| % car owners needing to install seatbelts or  % motor/bicyclists who are regular riders | 0% | 95% | 70% |
| Unit cost of seatbelt / helmet (annualised) (I$) | 8 | 2.4 | 1.2 |

Table 7: Total costs repartition in Southeast Asia

|  | **Pop° coverage** | **Individual level costs** | **Behaviour change communication costs** | **Programme support costs (including Passage of legislation and law enforcement costs)** |
| --- | --- | --- | --- | --- |
| Random breath testing | 80% | 0.00% | 2.28% | 97.72% |
| Speed limits | 80% | 0.00% | 2.22% | 97.78% |
| Bicycle helmet use | 80% | 0.46% | 2.39% | 97.14% |
| Motorcycle helmet use | 90% | 24.44% | 1.78% | 73.78% |
| Seatbelt use | 50% | 0.00% | 1.64% | 98.36% |
| Seatbelt use + Motorcycle helmet use | 90% | 22.32% | 3.25% | 74.42% |
| Seatbelt use + Motorcycle helmet use + Random breath testing | 90% | 20.18% | 4.41% | 75.40% |
| Seatbelt use + Motorcycle helmet use + Speed limits | 80% | 20.42% | 3.97% | 75.61% |
| Seatbelt use + Motorcycle helmet use + Speed limits + Random breath testing | 90% | 18.43% | 5.38% | 76.19% |
| Seatbelt use + Motorcycle helmet use + Speed limits + Random breath testing + Bicycle helmet use | 90% | 16.76% | 6.04% | 77.20% |
| Speed limits + Random breath testing | 80% | 0.00% | 3.84% | 96.16% |
| Speed limits + Random breath testing + Motorcycle helmet use | 90% | 20.14% | 4.41% | 75.45% |
| Speed limits + Random breath testing + Seatbelt use | 80% | 0.00% | 5.08% | 94.92% |

Table 8: Total costs repartition in Eastern sub-Saharan Africa

|  | **Pop° coverage** | **Individual level costs** | **Behaviour change communication costs** | **Programme support costs (including Passage of legislation and law enforcement costs)** |
| --- | --- | --- | --- | --- |
| Random breath testing | 80% | 0.00% | 2.02% | 97.98% |
| Speed limits | 80% | 0.00% | 2.01% | 97.99% |
| Bicycle helmet use | 80% | 0.07% | 2.04% | 97.89% |
| Motorcycle helmet use | 90% | 0.12% | 2.19% | 97.69% |
| Seatbelt use | 50% | 0.00% | 1.39% | 98.61% |
| Seatbelt use + Motorcycle helmet use | 90% | 0.11% | 3.84% | 96.05% |
| Seatbelt use + Motorcycle helmet use + Random breath testing | 90% | 0.10% | 5.11% | 94.79% |
| Seatbelt use + Motorcycle helmet use + Speed limits | 80% | 0.10% | 4.64% | 95.26% |
| Seatbelt use + Motorcycle helmet use + Speed limits + Random breath testing | 90% | 0.09% | 6.12% | 93.79% |
| Seatbelt use + Motorcycle helmet use + Speed limits + Random breath testing + Bicycle helmet use | 90% | 0.12% | 6.90% | 92.98% |
| Speed limits + Random breath testing | 80% | 0.00% | 3.51% | 96.49% |
| Speed limits + Random breath testing + Motorcycle helmet use | 90% | 0.09% | 5.11% | 94.80% |
| Speed limits + Random breath testing + Seatbelt use | 80% | 0.00% | 4.67% | 95.33% |
